# Physical Healthcare, Health-Related Quality of Life and Global Functioning of Persons with a Severe Mental Illness in Belgian Long-Term Mental Health Assertive Outreach Teams: A Cross-Sectional Self-Reported Survey

**DOI:** 10.3390/ijerph19095522

**Published:** 2022-05-02

**Authors:** Nicolaas Martens, Marianne Destoop, Geert Dom

**Affiliations:** 1 Multiversum Psychiatric Hospital, Brothers of Charity Belgium, 2530 Boechout, Belgium; marianne.destoop@uantwerpen.be (M.D.); geert.dom@uantwerpen.be (G.D.); 2 Collaborative Antwerp Psychiatry Research Institute (CAPRI), University of Antwerp, 2610 Antwerp, Belgium; 3 Department of Nursing, Karel de Grote University of Applied Sciences, 2018 Antwerp, Belgium

**Keywords:** physical health, severe mental illness, community mental health

## Abstract

Research shows that care delivery regarding somatic health problems for patients with a severe mental illness (SMI) in community and mental health is difficult to establish. During the last decade, long term mental health outreach teams in Belgium were implemented to provide treatment and follow-up at home. This study aimed to map physical health status, care professionals, health related quality of life and global functioning in persons with SMI in Belgian long term outreach teams for mental health. Using a self-administered questionnaire, 173 persons, 58.1% female with a mean age of 48.3, were questioned. Our findings suggest an undertreatment of somatic comorbid conditions, with only half of physical health complaints being addressed. Although treatment rates for hypertension, when detected were high, treatment of respiratory complaints, pain and fatigue was lacking. Although the majority of respondents responded to have a GP or psychiatrist, contact rates were rather limited. Other disciplines, such as primary care nurses, when present, tend to have more contact with people with SMI. Notably, having regular contacts with GPs seems to improve physical health complaints and/or treatment. Being treated by an outreach team did not show significant correlations with physical health complaints and/or treatment suggesting a more proactive approach by outreach teams or primary care providers is desirable.

## 1. Introduction

One of the greatest challenges in persons with a severe mental illness (SMI), such as schizophrenia, bipolar disorder or major depression, is tackling associated somatic comorbidities. These physical health comorbidities are responsible for a mortality gap with a standardized mortality rate 2.6 to 5 times higher when compared to the general population [1,2,3]. Sixty percent of this mortality can be attributed to physical health factors, such as being overweight, diabetes, cardiovascular conditions and respiratory health problems [4].

Worldwide, there is a growing public health concern regarding the topic of physical health disparities and somatic care in people with SMI, culminating in an increasing number of guidelines and tools, e.g., the guidelines elaborated by the World Health Organization (WHO) or the British National Institute for Care and Care Excellence (NICE) [5,6]. In Belgium, the governmental Health Care Knowledge Centre (KCE) recently published a guidance on somatic care for people with severe mental illness, but the recommendations provided by this guidance were limited to the context of a residential setting [7]. All of these guidelines recommend an integrated (somatic and mental health) care approach for persons with a SMI. However, in real life practice translation and implementation of these guidelines and principles remain very limited [8,9].

This lack of effective care delivery regarding somatic health problem for patients with SMI exists already for many years. Research shows that general practitioners (GPs) show lower stigmatization tendencies and more awareness in somatic care delivery for persons with a SMI if a GP is better interconnected with and supported by mental health services [10]. This is in line with patient perspectives, where separation of services are perceived as main barrier to care for people with SMI [11].

Previous research has established that health professionals’ alliances in providing simultaneous physical and mental health services to persons with a SMI are to the greatest extent formed by contacts between psychiatrists, (mental health) nurses and general practitioners. Integration of health care systems combined with outreach strategies directing medical somatic screening may lower barriers to care and increase health outcomes in persons with a SMI. Furthermore, a study in the United Kingdom (UK) showed that incentivizing physical health management in primary care for persons with a SMI increases screening and treatment rates, and could decrease premature mortality in persons with a SMI [12,13,14,15,16].

Physical health is strongly associated with the quality of life for individuals both in the general population as in people with SMI. However, people with a SMI are disproportionally touched by physical health problems. This, together with their mental health problems, impacts negatively their quality of life. In addition, poor physical health may limit global functioning, which could lead to unemployment, social isolation and low self-management. Further, the forementioned factors could contribute to social, economic and health deterioration [1,4,17,18].

Using a cross-sectional patient survey, this study attempts to identify professional caregivers involved in physical health care for people with a SMI treated by long-term mental health community outreach teams in Belgium.

The primary aim of this study is to explore whether the number of professionals involved, the type of health care professionals and the number of health care contacts could be related to physical health complaints and treatment, Health Related Quality of Life (HR-QOL) and global functioning.

It concerns an exploratory study that could contribute into a further understanding of possible influential factors on physical health complaints in persons with a SMI, and could be of added value in developing a tailored integration of health care delivery adapted to the Belgian. It is beyond the scope of this study to examine causal effects.

## 2. Materials and Methods

A cross-sectional, quantitative method was used to investigate physical complaints of persons with a SMI (in contact with a long-term mental health (MH) outreach team), their experienced HR-QOL and global functioning. Also, a stakeholder mapping was performed to identify professional (i.e., general practitioner; primary care nurse; pharmacist; psychologist; psychiatrist) caregivers. In addition, respondents were asked to estimate the frequency of contacts for each caregiver.

### 2.1. Participants

Using a convenience sampling method all clients of 8 long-term MH-outreach teams in the Flemish region of Belgium were invited by their personal case manager to complete the questionnaire. Exclusion criteria were: being intellectually disabled; no understanding of the Dutch language and having acute psychiatric symptoms (i.e., being unable to complete the questionnaire due to acute psychotic, depressive/manic or behavioral symptoms, assessed by the outreach team).

The survey was originally initiated within 4 MH-outreach teams (teams 1–4) in February 2020 but, due to the COVID-19 pandemic, paused between March 2020 and September 2020. To ensure a representative sample size, 4 additional teams (teams 5–8) were added in February 2021. These 8 teams represent about 25% of the teams (*n* = 31) covering the whole of the Flemish region.

The questionnaires were distributed by the case managers in the outreach team. Each case-manager handed over an information form and the questionnaire to all of the patients in their personal caseload if patients were eligible for inclusion. After completing the questionnaire, patients returned the survey in a closed envelop to the case manager. If requested by the patient, a verbatim questionnaire could be performed by the case manager.

### 2.2. Measures

A self-administered paper structured questionnaire was used to assess following items:

Demographic data:

- Age
- Gender: Man; woman; undefined.
- Living situation: Living alone; living together with spouse.
- Number of months treated by the mental health outreach team
- Psychiatric Diagnosis; schizophrenia; depression; bipolar disorder; borderline personality disorder; psychosis; was never diagnosed; other.
- Informed of psychiatric diagnosis by: Psychiatrist; psychologist; general practitioner; nurse; was never informed; other.
- Living area (Rural, suburban or urban)

Health related quality of life (HR-QOL):

To assess HR-QOL, the Dutch version of the RAND-36 item health survey was used [19]. The RAND-36 is a widely used instrument comprised of 36 items that assesses eight health domains: physical functioning, role limitations caused by physical health problems, role limitations caused by emotional problems, social functioning, emotional well-being, energy/fatigue and pain. In addition, he RAND-36 also provides a score for general health perceptions and summary scores for physical and mental health [20,21]. The RAND-36 is based on the MOS 36-Item Short-Form Health Survey (SF-36) and includes the same items as those in the SF-36, but the recommended scoring algorithm is somewhat different from that of the SF-36 [21]. RAND-36 reliability is high and Cronbach’s Alpha varies between 0.81 and 0.95 [22,23].

Global Functioning:

Global functioning was evaluated using the World Health Organization Disability Assessment Schedule (WHODAS 2.0), a questionnaire recommended by the DSM-5 Disability Study Group to estimate global functioning in people with a psychiatric disorder. A literature review also mentioned an increasing interest in the WHODAS 2.0 to assess individual global functioning and disability in different types of settings and individual health conditions. disability [24,25,26]. The Dutch version of the WHODAS 2.0 scale is validated and considered reliable (Cronbach’s alpha = 0.98) and applicable in people with a SMI, both in residential as community settings [27,28].

Physical health status:

Respondents were asked to indicate which physical health complaints they experienced and for which health problems they already received treatment. Using a structured questionnaire, respondents were asked to indicate the forementioned using following categories: Respiratory health; high blood pressure; gastro-intestinal health; being overweight; tiredness and pain. Using this information, the total number of health complaints was calculated.

Stakeholder mapping:Care professionals surrounding the respondents were explored, by asking respondents to report following items:

- Type of professional caregivers present in the patients’ care network: General practitioner; psychiatrist; pharmacist; psychologist; home or primary care nurse; other.
- Type of professional caregiver with whom the patient has at least a monthly contact: General practitioner; psychiatrist; pharmacist; psychologist; home or primary care nurse; other.

To explore the balance between their personal and professional network, respondents were asked to divide 100% of the contacts they had encountered during the past month. Respondents were asked to distribute 100% between care professionals and their own social network (other social contacts excluding health- or welfare professionals).

Medication management:

Respondents were requested to indicate who is responsible for their medication management in general, consisting of medication distribution, conservation and administration. Respondents could make a choice of one or multiple of following choices: Myself; family, pharmacist; home nurse; outreach team; other.

### 2.3. Data Analyses

Statistical analyses were performed using Statistical Package for the Social Sciences (SPSS), version 28 (IBM^®^, Armonk, NY, USA, 2021). Using Mann-Whitney tests, significant differences between dichotomous variables (gender, having a type of professional caregiver or not, having regular contacts with a specific caregiver or not) on outcome variables HR-QOL, global functioning and physical health complaints were analyzed. Kruskal Wallis tests were used to explore significant differences between:

- Diagnosis groups (schizophrenia; depression; bipolar disorder; borderline personality disorder; psychosis; was never diagnosed; other) in number of physical health complaints and treatment.
- The included teams for respondents’ age and length of treatment by the outreach team.
- The total number of professional caregivers and RAND-36 MCS, RAND-36 PCS, and WHODAS 2.0.
- The number of type of care professional respondents reported to have regular contacts with in RAND-36 MCS, RAND-36 PCS and WHODAS 2.0 scores.
- Using Chi-square tests, possible differences between the included teams regarding psychiatric diagnosis or living area were explored.

Using the Spearman’s correlation coefficient, possible relations between number of caregivers; number of contacts with caregivers; duration of care outreach team and number of physical health complaints (being treated) were examined. For all analysis, the significance level was set on *p* < 0.05.

## 3. Results

### 3.1. Demographic Data

A total of 173 patients completed the questionnaire. Demographic data is listed in Table 1.

Age among respondents was not normally distributed (Shapiro Wilk test, *p* = 0.009) but slightly negatively skewed (skewness −0.39; kurtosis −0.47), duration of the treatment also was not normally distributed (Shapiro Wilk test, *p* < 0.001). Scale scores however were normally distributed (Shapiro-Wilk test: RAND-36 physical health *p* = 0.41; RAND-36 mental health *p* = 0.42; WHODAS 2.0 = 0.33). Considering data was not normally distributed, and small group sizes when comparing groups, non-parametric statistical tests were used during analyses, and central tendencies were displayed using median and interquartile range (IQR). When comparing the included outreach teams, Kruskal-Wallis tests showed significant differences regarding distribution of the patients’ median age (Χ^2^ = 14.97; *p* = 0.036; Mean rank scores: Team 1 = 96.57; Team 2 = 65.39; Team 3 = 73.89; Team 4 = 120.50; Team 5 = 78.58; Team 6 = 92.21; Team 7 = 73.72; Team 8 = 106.64) and length of treatment by the outreach teams (Χ^2^ = 27.57; *p* < 0.001; Mean rank scores: Team 1 = 97.57; Team 2 = 68.13; Team 3 = 60.50; Team 4 = 67.00; Team 5 = 52.68; Team 6 = 76.92; Team 7 = 89.98; Team 8 = 112.08). However, pairwise comparisons between teams using Bonferroni correction for multiple tests yielded no specific significant differences between teams for mean age and length of treatment by the outreach teams (data not displayed).

Chi-square tests showed significant differences between the included teams in both psychiatric diagnosis (Χ^2 =^ 76.99; *p* = 0.001) and living area (Χ^2^ = 112.12; *p* < 0.001). Percentages per team are displayed in Table 2.

### 3.2. Physical Health and Professional Care Network

The median number of physical health complaints respondents experienced was 3 health complaints (IQR = 2). The median number of complaints respondents reported to receive treatment for was 1(IQR = 4). An overview of reported type of complaints and within group treatment rates are presented in Table 3. Using Kruskal Wallis tests, no significant differences were found between diagnosis groups for number of physical complaints (Χ^2^ = 8.53; *p* = 0.07; Mean rank scores: Psychotic disorder = 63.57; Mood disorder = 78.90; Personality disorder = 94.17; Dual diagnosis = 89.59). Also, for receiving treatment for health complaint(s) no significant differences were found between diagnosis groups (Χ^2^ = 7.20; *p* < 0.13; Mean rank scores: Psychotic disorder = 67.05; Mood disorder = 76.51; Personality disorder = 95.02; Dual diagnosis = 71.27).

22% of all respondents mentioned having health complaints within the category ‘other’. Within this category respondents reported following health complaints: Cardiac (*n* = 9), hormonal (*n* = 6), orthopedic (*n* = 6), neurological (*n* = 5) and diabetic (*n* = 3) health problems.

**Figure 1:**
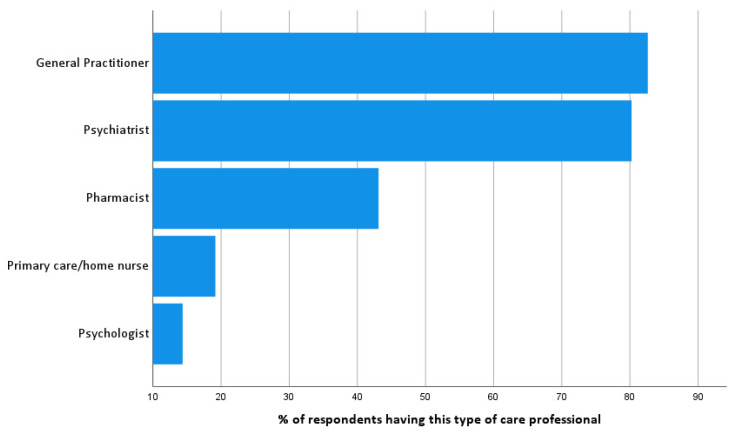
Type of care professionals being present in the respondents’ personal care network (% of respondents).

To identify professional stakeholders, respondents were asked to list the professional caregivers they are in contact with (Figure 1). When present, respondents tend to have most contacts with their psychologist (91.7%), followed by a primary care home nurse (83.9%), psychiatrist (67.7%), pharmacist (67.6%) and the general practitioner (50.4%). Off all of the respondents’ contacts, 51.3% were personal social contacts and 48.7% were contacts with health professionals.

A Mann-Whitney U test was used to analyze possible significant differences between having a specific type of caregiver or not on the number of physical health complaints and the number of health complaints being treated. The same procedure was used regarding any differences in having regular contacts with specific care professionals. Results are shown in Table 4.

In terms of medication management 80.7% of the respondents registered that they are personally responsible for management of medication; 14.5% are supported by the pharmacist; 12.7% by primary care home nurses; 7.8 by family. In 14.9% of the cases a combination of different possibilities is being used. The assertive outreach team or personal case manager was never involved concerning medication management.

### 3.3. Health Related Quality of Life (RAND-36)

**Figure 2:**
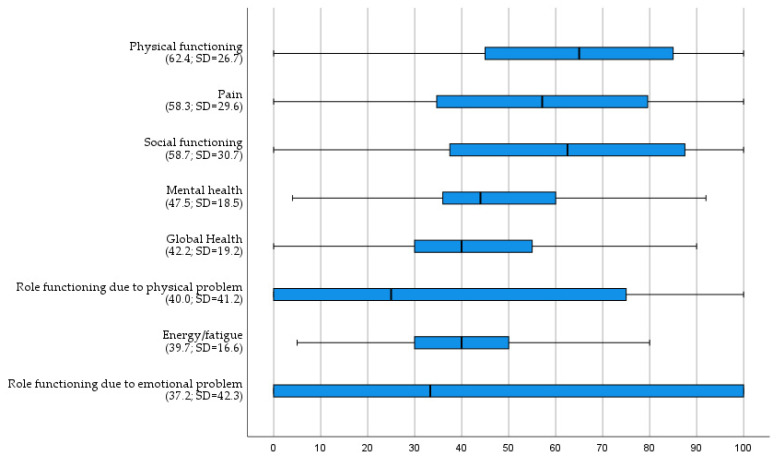
Boxplots of RAND-36 subitems. (low scores indicate a higher impairment per item).

RAND-36 subscales were calculated and are shown in Figure 2. Scores on role functioning due to a physical or emotional problem scored lowest. When comparing the differences on RAND-36 subscale scores using a Mann-Whitney U test between having a specific type of professional caregiver or not, having a general practitioner showed a significant lower score on ‘role functioning due to an emotional problem’ (U = 1389.5; *p* = 0.03). Having a primary care home nurse showed a significant lower score on the subscale ‘physical functioning’ (U = 1407.5; *p* = 0.02).

Using Mann-Whitney U tests, differences on the RAND-subscales depending on having regular contacts with a specific type of care professional or not yielded significant lower scores for contact with a psychologist on the subscale ‘social functioning’ (U = 1939; *p* = 0.03), having regular contacts with a pharmacist showed significant lower scores on ‘physical functioning’ (U = 2547.5; *p* = 0.03) and ‘role functioning due to a physical problem’(U = 2386.5; *p* < 0.001). Having regular contact with a primary care home nurse showed significant lower scores on ‘physical functioning’ (U = 1256.5; *p* = 0.008) and ‘pain’ (U = 1549; *p* = 0.04). Having regular contacts with a general practitioner resulted in the highest number of significant differences, with lower scores on subscales ‘physical functioning’ (U = 1977.5; *p* < 0.001), ‘social functioning’ (U = 2790; *p* = 0.03), ‘role functioning due to a physical problem’ (U = 2167.5; *p* < 0.001), ‘mental health’ (U = 2664.5; *p* = 0.02), ‘pain’ (U = 2113; *p* < 0.001) and ‘global health’ (U = 2407.5; *p* = 0.01).

RAND-36 scores showed a good internal consistency (Cronbach’s Alpha = 0.85). Total mental health (MCS) and physical health component scales (PCS) were calculated using following 3 steps proposed by Ware (1995): create standardized z-scores for each subscale; aggregating data into a standardized mental and physical health component scale; perform T-score transformation (50 + (10 × standardized component scale)) per standardized component scale. The median PCS was 49.7 (IQR = 15.7), median MCS was 49.2 (IQR = 14.3). Mann-Whitney U tests were used to compare PCS and MCS between men and women. Although no significant difference were found between men and women on PCS (50.4 (IQR = 12.5) vs. 48.7 (IQR = 17.8; U = 2460; *p* = 0.36), median scores of men on the MCS were significantly higher compared to women (52.9 (IQR = 14.0) vs. 47.8 (IQR = 14.4); U = 2187 *p* = 0.05) (Data not shown in this article).

### 3.4. Global Functioning (WHODAS 2.0)

Reliability analysis showed good consistency (Cronbach’s Alpha = 0.83). The median total score for the WHODAS 2.0 scale was 40.2 (IQR = 18). Because only 12% of the respondents did paid or voluntary labor, the total score was calculated excluding work items. Following median scores for the subscales were reported (higher score on the WHODAS 2.0 scale corresponds with a higher level of disability): ‘Participation in society’ (50 (IQR = 33)); ‘Household activities’ (40 (IQR = 40)); ‘Getting along with people’(41.7 (IQR = 46)); ‘Mobility’(37.5 (IQR = 46)); ‘Understanding and communicating’(35 (IQR = 35)) and ‘Self-Care’(20 (IQR = 30)).

### 3.5. Correlational Analyses

Using the Spearman rank-order correlation, significant but low correlations were identified between ‘Number of physical complaints’ and the amount of contacts with family/friends (r = −0.18, *p* = 0.02) and having regular contacts with caregivers (r = 0.26, *p* < 0.001). The variable ‘Number of physical health complaints being treated’ was significantly correlated with the amount of contacts with friends and family (r = −0.22, *p* = 0.004) and having regular contacts with caregivers (r = 0.23, *p* = 0.003). Both ‘number of physical health complaints’ (r = 0.02; *p* = 0.78) and ‘number of complaints being treated for’ (r = 0.04; *p* = 0.60) were not significantly correlated with the duration of treatment by an outreach team. Duration of treatment by an outreach team and ‘number of professional caregivers’ showed a low significant correlation (r = 0.16; *p* = 0.04). Significant correlations particularly concerning care professionals and physical health complaints are shown in Table 5.

### 3.6. Professional Caregivers, RAND-36 and WHODAS 2.0 Outcomes

To evaluate possible differences between the total number of professional caregivers and RAND-36 MCS, RAND-36 PCS, and WHODAS 2.0 scores Kruskal-Wallis tests were performed. Results showed no significant differences between the number of professional caregivers in the personal care network and:

1. RAND-36 MCS (X^2^ = 1.32; *p* = 0.86; Mean rank scores: 0 Caregivers = 70.38; 1 Caregiver = 83.39; 2 Caregivers = 77.47; 3 Caregivers = 73.07; 4 Caregivers = 69.95)
2. RAND-36 PCS (X^2^ = 8.08; *p* = 0.089; Mean rank scores: 0 Caregivers = 89.88; 1 Caregiver = 71.11; 2 Caregivers = 79.14; 3 Caregivers = 79.36; 4 Caregivers = 51.95)
3. WHODAS 2.0 total score (X^2^ = 5.80; *p* = 0.22; Mean rank scores: 0 Caregivers = 66.50; 1 Caregiver = 65.21; 2 Caregivers = 66.02; 3 Caregivers = 75.36; 4 Caregivers = 91.37).

Using Kruskal Wallis tests, possible differences between the number of professional caregivers having regular contacts with the respondents on RAND-36 MCS, RAND-36 PCS and WHODAS 2.0 scores were assessed. No significant differences were found regarding:

1. RAND-36 MCS (X^2^ = 4.83; *p* = 0.31; Mean rank scores: 0 Caregivers = 89.05; 1 Caregiver = 64.42; 2 Caregivers = 76.00; 3 Caregivers = 73.89; 4 Caregivers = 67.29)
2. WHODAS 2.0 (X^2^ = 8.36; *p* = 0.07; Mean rank scores: 0 Caregivers = 58.91; 1 Caregiver = 59.63; 2 Caregivers = 77.22; 3 Caregivers = 84.21; 4 Caregivers = 69.53).

RAND-36 PCS showed significant differences between the number of care professionals with whom patient had regular contacts (X^2^ = 21.21; *p* < 0.001; Mean rank scores: 0 Caregivers = 90.64; 1 Caregiver = 97.71; 2 Caregivers = 66.81; 3 Caregivers = 56.49; 4 Caregivers = 63.06).Post-hoc pairwise comparison showed, after Bonferroni correction, a significant different RAND-36 median PCS score between having regular contacts with: 3 vs. 0 caregivers (median PCS 44.01 vs. 55.62; X^2^ = 34.50; *p* = 0.05); 3 vs. 1 caregiver (median PCS 44.01 vs. 57.64; X^2^ = 41.73; *p* = 0.001); 2 vs. 1 caregiver (median PCS 47.88 vs. 57.64; X^2^ = 31.36; *p* = 0.03).

## 4. Discussion

The present study was designed to explore whether the number of professionals involved, the type of health care professionals and the number of health care contacts could be related to physical health complaints and treatment, HR-QOL and global functioning in persons with a SMI. Prior studies have highlighted the importance of the management of physical health in persons with a severe mental illness in decreasing premature mortality [29,30]. To our best knowledge however, to this date no study has examined the prevalence of and number of contacts with professional caregivers and the potential relation with physical healthcare delivery in persons with a SMI.

### 4.1. Physical Health and Care Professionals

Results of this study suggest that of all patients within a MH-outreach team, around 80% reported to have a GP and a psychiatrist. Notably, around 50% of the patients reported to have regular contacts with their GP and/or psychiatrist. This could be regarded as rather high, as a recent Belgian governmental report stated that, of all people with mental health problems, only 30% consults a GP, and 43% a GP and a psychiatrist. However, it needs to be underlined that this report targeted the number of contacts because of mental health complaints, and therefore differs from this study which targets contacts because of physical health complaints [31]. As our study showed that half of the respondents reported to have regular contacts with their GP, this may suggest that treatment by an MH-outreach team can improve GP involvement when compared to this governmental report. Nevertheless, no significant relation was found in duration of treatment by an outreach team and physical health outcomes.

In our study, respondents reported to have approximately 3 health complaints compared to 1 complaint for which they receive treatment. This underlines a possible gap in adequately addressing physical health disparities given the fact that merely 50% of physical health complaints are being addressed. Although reported treatment rates for high blood pressure were high, other complaints remained rather undertreated. In particular, treatment rates for respiratory health complaints, overweight and fatigue were low. This finding has important implications for developing future health care services, because both cardiovascular and respiratory health complaints contribute vastly to excessive mortality due to physical health in persons with SMI [32]. Screening of cardiovascular risk is a cost-effective routine practice in primary care in the global population, and therefore it should be feasible to tailor interventions to persons with a SMI. Chronic Obstructive Pulmonary Disease (COPD), the most prevalent respiratory condition in persons with a SMI, is often underdiagnosed in the general population. Specifically for persons with SMI, it is estimated that approximately 8% of all persons with COPD have a SMI, but quality of care is lower compared to the general population [33,34,35].

All the respondents in this study received treatment by a MH-outreach team. No significant correlations between the duration of treatment by an MH-outreach team and number of physical health complaints or number of complaints being treated were found. This finding is in line with our previous research suggesting that Belgian MH-outreach teams have little attention for somatic comorbidities, and the actual treatment has a more psychosocial approach [36]. This is an important implication for developing a more systematic approach by MH-outreach teams towards physical health disparities in persons with a SMI to improve physical health consultations and screening rates in primary care [13,31].

The number of professional caregivers involved showed a significant positive correlation with the number of physical health complaints and health complaints being treated. This carefully may imply a more reactive approach towards physical health complaints in persons with a SMI instead of targeting prevention. However, a more preventive approach is recommended in persons with a SMI, as treatment of somatic comorbidities such as cardiovascular conditions, should commence at a younger age compared to the general population [37].

Respondents had, when represented in their personal care network, most contacts with primary care nurses and psychologists. However, primary care nurses and psychologists were only represented in respectively 19% and 14% of the respondents’ professional care network. This can be perceived as an opportunity in targeting physical health disparities in a more effective way, as nurses could plan and implement interventions to address lifestyle, somatic screening and follow-up. This could potential result in significant decreases in waist circumference, improvement in lifestyle and physical functioning or HR-QOL [38].

Merely 51.2% of the respondents reported to be surrounded by friends and/or family. In addition, our study found a small but significant negative correlation between the number of contacts with family/friends and both the number of physical health complaints and physical health complaints being treated, carefully suggesting that having less social contacts could impede psychical health outcomes or vice versa. However, literature suggests it could be beneficial to involve central persons within the patients’ personal network to improve health consultations [39,40]. It needs to be mentioned that, in community mental health care, although informal caregivers could affect patient outcomes positively, involving informal caregivers often seems not feasible in people with SMI. A recent cross-sectional study suggested that 54.7% of the family caregivers reported experiencing loneliness and a low QOL themselves. Social support is needed to improve care burden and loneliness among family caregivers [39,41].

Concerning medication management, this study shows that 43% of the respondents has a pharmacist, but only 14.5% is supported by the pharmacist for medication management. However literature regarding the role of the pharmacist in community (mental) healthcare is rather scarce, a review shows positive effects on cardiometabolic outcomes when a more collaborative approach including pharmacies is established. Pharmacists could provide individual education and referrals to people with SMI, and support professionals in community mental health care as members of multidisciplinary teams [42]. Currently, in real-life community settings, integration of pharmaceutical care for persons with a SMI in is often impeded by medical services that are office-based psychiatrists or nursing services reimbursed per specific performance, which is a drawback, as patients’ knowledge and attitudes about health conditions and pharmaceutical treatment positively affects their self-management [43,44,45]

### 4.2. Quality of Life

Although the Dutch version of the RAND-36 is a widely used questionnaire to assess HR-QOL in persons with various physical conditions, research concerning people with SMI in Belgium is rather scarce [20]. When comparing mean RAND-36 scores to existing literature, results in our study are systematically lower compared to equivalent samples of patients with SMI: Physical functioning (62.4 vs. 76.6–79.5); Physical role functioning (40 vs. 68.8–76.8); Pain (58.3 vs. 67.2–84.2); Global Health (42.2 vs. 56.3–63.4); Social Functioning (58.7 vs. 77.7–79.3); Emotional Role functioning (37.2 vs. 56.3–73.2); Energy/fatigue (39.7 vs. 47.2–67.6); Mental Health (47.7 vs. 63.3–68.14) [46,47,48]. Although the study design impedes identifying a clear reason to explain this differences remains unclear, organization of Belgian community mental health care could be an explanatory factor. Firstly, compared to other countries only 6% of the global health budget in Belgium is invested in mental health services, which is low compared to other European countries [49]. Secondly, Belgian outreach teams in Belgium primarily provide psychosocial support, and often are composed of non-medical professionals. Countries with a higher HR-QOL in people with SMI often use more integrated approaches provided by nurse practitioners [36,50]. Overall, persons with a severe mental illness tend to score lower on HR-QOL compared to the general population [51].

MH-outreach teams should actively built collaborations with GP’s and primary care nurses. Physical health screening and follow-up should be a formal part in building collaborations, as previous research showed that neglecting physical health in building collaborations could be a pitfall [43]. HR-QOL related to physical health can be influenced by installing more interventions targeting physical health more directly, using peer-navigators, integration of services or liaison services [52].

Surprisingly, when present contact rates with psychiatrists were rather high (67.7%). However, the present studyfound no significant differences on any subscale or total RAND-36 scores for having regular contacts with a psychiatrist.

### 4.3. Global Functioning

Comparing results with a recent study combining Central and East European data in 931 persons with a severe mental illness assessing functional limitations using the WHODAS 2.0, similar results were found in our study (total score 21.5–37.1), including the highest degree of impairment in the domains ‘Participation in society’, ‘Household activities’ and ‘getting along with people’ [53]. Another study comparing inpatients and outpatients in a Caribbean region showed lower scores on all subscales and the total score (38.3 for outpatients), but outpatients also scored highest on participation in society [27]. It needs to be mentioned that, when compared to WHODAS 2.0 general population norms, respondents of the present study are placed between percentile 85.8 and 90.4 of the total population norm. This indicates a high level of functional disability in persons with a SMI [28]. Although the WHODAS 2.0 is well validated and reliable, a lack of comparable Belgian data however limits a profound interpretation of the results within the present study.

Regarding physical health, creating routine, structure and planning for persons with SMI can promote habit formation and minimize disability, influencing both positive and negative health behaviors [45]. This method can be useful in addressing the low scores on the subscales ‘Understanding and Communication’ measured in our study, so improving access to specialist services in terms of health behavior may be beneficial.

Overall, additional staff to decrease workload and share knowledge may be valuable, in combination with increasing time for appointments in primary care for people with SMI to meet patient needs [54]. A holistic approach targeting both mental and physical health care using patient-driven treatment plans is recommended. In facilitating sustainable collaborations between primary care and mental health care services, integration should be established tailored to the local context and to patients’ and stakeholders’ needs [55,56].

Specific for the Belgian Mental Healthcare Reform, research shows that both patients and staff complacency of care delivered by Belgian outreach teams is described as being good [39]. However, collaboration rates within mental health services itself mostly depend on informal contacts and agreements and there is a need for more formalized collaboration [57]. This could arise the assumption that methods of collaboration regarding physical health for persons with SMI, formed outside mental health services itself do not rely on well-structured formal agreements.

### 4.4. Limitations

Given the fact that physical health disparities in persons with SMI continues to be a difficult topic to address in practice, our study aimed to gain a further insight in this issue.

It is important to be aware of the predictive limitations of cross-sectional studies, and results should be interpreted with caution. In particular one should be careful in generalizing the findings. Despite the limitations of the exploratory nature of this study it offers some insight in addressing physical health complaints and HR-QOL. Notwithstanding the relatively limited sample, this work also adds to the knowledge of Belgian community mental health outreach services in the backcloth of the Belgian Mental health care reform. Although the theoretical implications of these findings are unclear, this study suggests that physical health complaints in persons with a SMI in Belgium could remain undetected and untreated, and possibly have an influence on HR-QOL and global functioning. A more active role of a MH-outreach team in screening and follow-up of physical health needs could be beneficial in establishing appropriate collaborations to minimize physical health disparities in people with SMI.

As this study is limited to merely evaluating numbers of caregivers and number of physical health complaints and HR-QOL in a quantitative exploratory manner, a more qualitative in depth approach is necessary to adequately interpret the findings of this study and to provide further insights in improving physical health and contribute to minimizing physical health inequality in a Belgian context.

## 5. Conclusions

This study evaluated physical health, HR-QOL and global functioning in persons with SMI in Belgian long term outreach teams for mental health, and explored possible relations with professional caregivers in the patients’ care networks. Results suggest an undertreatment of somatic comorbid conditions, in particular respiratory complaints, pain and fatigue. Although the majority of respondents responded to have a GP or psychiatrist, contact rates were rather limited. Pharmacists were not reported as a caregivers by most respondents, and contacts were limited. Other research concerning the role of the pharmacist was limited but pharmacists could be of added value in addressing physical health disparities. Scale scores regarding HR-QOL were lower compared to comparable research, specific reasons for this findings however remain unclear. Regarding global functioning, respondents scored between percentile 86 and 90 of the general population, indicating a vast functional impairment amongst respondents of this study. Relations between the number of professional caregivers, QOL and global functioning were mostly not significant Being treated by an outreach team also did not show significant correlations with physical health complaints and/or treatment. As this study design has many limitations, no results can be generalized. Other research mentions possible positive effects of including family, friends and primary care home nurses in improving physical health care, but is rather limited and unclear. To adequately understand and interpret these findings and further explore integrated care approaches targeting physical health in people with SMI, complementary qualitative research is needed.

## Figures and Tables

**Table 1:** Demographic data of respondents included in the study.

	Age (Median(IQR))	Gender (%)	Psychiatric Diagnosis (% of Respondents)	Number of Months Treated by Team (Median(IQR))	Living Area (% of Respondents)
*n* = 173	50(18)	58.1% Female 41.9% Male	25.4% Psychotic disorder 41.4% Mood disorder 13% Personality disorder 7.1% Substance abuse 6.5% Dual Diagnosis substance abuse/psychosis 4.7% Other 1.8% Unknown	24(36)	48.8% Rural 34.9% Suburban 16.3% Urban

**Table 2:** Percentual distribution of patients’ living area and diagnosis groups within each team.

		Team							
		1	2	3	4	5	6	7	8
Living Area	Urban	0.0%	13.6%	89.5%	0.0%	0.0%	26.9%	0.0%	3.6%
	Suburban	41.4%	22.7%	5.3%	33.3%	35.0%	11.5%	68.0%	50.0%
	Rural	58.6%	63.6%	5.3%	66.7%	65.0%	61.5%	32.0%	46.4%
Diagnosis Group	Psychotic disorder	51.7%	22.7%	15.8%	0.0%	10.0%	16.0%	8.0%	46.2%
	Mood disorder	41.4%	59.1%	36.8%	33.3%	40.0%	44.0%	36.0%	34.6%
	Personality disorder	3.4%	13.6%	10.5%	0.0%	15.0%	16.0%	24.0%	11.5%
	Substance abuse	0.0%	4.5%	15.8%	0.0%	10.0%	12.0%	8.0%	3.8%
	Dual Diagnosis substance abuse/psychosis	0.0%	0.0%	5.3%	33.3%	5.0%	8.0%	20.0%	3.8%
	Other	3.4%	0.0%	10.5%	0.0%	15.0%	4.0%	4.0%	0.0%

**Table 3:** Prevalence of physical health complaints among respondents.

Type of Physical Complaints	Respondents with Physical Health Complaints	Within Group Percentage Receiving Treatment for This Type of Complaint
Respiratory problems	37.7%	35%
High blood pressure	22.7%	94.1%
Overweight	44.9%	26.7%
Gastro-intestinal	40.1%	65.7%
Pain	34.1%	49.1%
Feeling tired	68.9%	23.5%

**Table 4:** Differences in number of physical health complaints/number of health complaints treated, compared between having a particular health care professional or not, and having regular (at least monthly) contacts with a particular health care professional or not (Median values + Inter-quartile range (IQR); U-value Mann-Whitney U test; * = significant Mann-Whitney U test).

	Number of Physical Health Complaints (Median(IQR))	Number of Physical Health Complaints Treated (Median(IQR))
General practitioner vs. No general practitioner	3(2) vs. 2.0(2) U = 1342.5; *p* = 0.005 *	1(4) vs. 1(1) U = 1507.5, *p* = 0.029 *
Regular contacts with general practitioner vs. No regular contacts with general practitioner	3.0(2) vs. 2(2) U = 2541.5; *p* = 0.003 *	1(4) vs. 1(1) U = 2430.5; *p* < 0.001 *
Psychiatrist vs. No psychiatrist	3(2) vs. 3(2) U = 2184.5; *p* = 0.91	1(4) vs. 1(2) U = 2194.5; *p* = 0.94
Regular contacts with psychiatrist vs. No regular contacts with psychiatrist	3(2) vs. 2(3) U = 2895; *p* = 0.10	1(2) vs. 1(4) U = 3183; *p* = 0.49
Pharmacist vs. No pharmacist	3(2) vs. 3(3) U = 3247; *p* = 0.57	1(4) vs. 1(1) U = 2906.5; *p* = 0.08
Regular contacts with pharmacist vs. No regular contacts with pharmacist	3(2) vs. 2(3) U = 2756.5; *p* = 0.02 *	1(4) vs. 1(2) U = 2633; *p* = 0.006 *
Primary care home nurse vs. No primary care home nurse	3.0(2) vs. 3(3) U = 1808.5; *p* = 0.14	1(2) vs. 1(2) U = 1988.5; *p* = 0.47
Regular contacts with primary care nurse vs. No regular contacts with primary care nurse	3(2) vs. (2.75) U = 1812; *p* = 0.33	1(2) vs. 1(2) U = 1892.5: *p* = 0.53
Psychologist vs. No psychologist	2.9(1.5) vs. 2.6(1.5) U = 1569; *p* = 0.49	1.0(0.9) vs. 1.2(1.1) U = 1572.5; *p* = 0.49
Regular contacts with psychologist vs. No regular contacts with psychologist	3(2) vs. 3(3) U = 2223.5; *p* = 0.25	1(2) vs. 1(2) U = 2450.5; *p* = 0.78

**Table 5:** Correlations between number of physical health complaints, physical health complaints being treated, number of care professionals having regular contact with patient and number of care professionals involved (Spearman’s rank correlation coefficient and significance level, * = significant correlation).

	Number of Patients’ Care Professionals Having Regularly Contact	Number of Physical Health Complaints Being Treated	Number of Physical Health Complaints	Number of Care Professionals in Patient’s Care Network
Number of patients’ care professionals having regularly contact	/	r = 0.23 *p* = 0.003 *	r = 0.26 *p* < 0.001 *	r = 0.48 *p* < 0.001 *
Number of physical health complaints being treated	r = 0.23 *p* = 0.003 *	/	r = 0.65 *p* < 0.001 *	r = 0.11 *p* = 0.14
Number of physical health complaints	r = 0.26 *p* < 0.001 *	r = 0.65 *p* < 0.001 *	/	r = 0.15 *p* = 0.054
Number of care professionals in patient’s care network	r = 0.48 *p* < 0.001 *	r = 0.11 *p* = 0.14	r = 0.15 *p* = 0.054	/

## Data Availability

University of Antwerp, data curator is Geert Dom.

## Glossary

COPD	Chronic Obstructive Pulmonary Disease
HR-QOL	Health related quality of life
IQR	Interquartile Range
KCE	Belgian Health Care Knowledge Centre
MCS	Mental Health Component Scale
MH	Mental Health
MOS	Medical outcome study
NICE	National Institute for Care and Care Excellence
PCS	Physical Health Component Scale
QOL	Quality of Life
RAND-36	36-item short form health survey
SF-36	36-item short form health survey
SMI	Severe Mental Illness
UK	United Kingdom
WHO	World Health Organization
WHODAS	World Health Organization Disability Assessment Schedule
